# Understanding the desire for hastened death in terminal cancer: Implications for whole-person palliative care

**DOI:** 10.1017/S147895152610265X

**Published:** 2026-05-04

**Authors:** Beulah Joy Damasco, Hazel Agbayani, Giovanni Jan Sanchez

**Affiliations:** College of Nursing, Bukidnon State University, Malaybalay City, Philippines

Dear Editor,

We have read with great interest the article by Matsumura et al. exploring the underlying structural mechanisms and whole-person perspectives on the desire for hastened death (DHD) in patients with terminal cancer. By emphasizing the multifaceted and relational nature of DHD through 6 interrelated themes such as loss of self-control, loneliness, perceived burden, and unwillingness to accept life changes, the authors provide a current and significant contribution. Their qualitative findings highlight the fact that DHD arises from intricate interactions in the psychological, social, and spiritual realms and goes beyond physical symptoms. For doctors managing existential discomfort in end-of-life care, this whole-person concept is very helpful (Matsumura et al. 2026).

The study supports earlier research showing that DHD is more strongly linked to multidimensional suffering and overall anguish than to a straightforward desire to die. Previous studies have demonstrated a high correlation between DHD in patients with advanced cancer and physical discomfort, sadness, pessimism, and loss of dignity (Chochinov et al. 1995; Breitbart et al. 2000; Chochinov 2023). In a similar vein, systematic reviews characterize DHD as a dynamic reaction to perceived loss of control, autonomy, and meaning that is influenced by existential and relational issues (Monforte-Royo et al. 2011; Borges et al. 2024). These results support the theory put forth by Matsumura et al. that DHD arises via structural pathways including temporality, autonomy, and relationality, which eventually result in existential meaninglessness and emotions of worthlessness.

But there are certain methodological issues that need to be clarified. First, the study only considers the viewpoints of palliative care providers, which might not accurately represent patients’ actual experiences. According to earlier qualitative research, patients’ accounts of DHD may not match physicians’ perceptions, especially when it comes to autonomy, dignity, and perceived burden (Rodríguez-Prat et al. 2017). Including in-person patient interviews could improve credibility and expand knowledge of the subjective definition of DHD. Second, generalizability outside of the study environment may be limited by cultural variables mentioned in the paper, such as relational identity and worries about being a burden. The necessity for comparative research across varied cultures is highlighted by cross-cultural examinations that show variation in how autonomy, family roles, and dignity shape DHD.

Notwithstanding these drawbacks, the study emphasizes consequences that are clinically significant. Research indicates that addressing existential suffering through relational support, communication, and care that preserves dignity may lessen DHD and enhance quality of life (Breitbart et al. 2000; Rodríguez-Prat et al. 2017). Emerging whole-person palliative care models that emphasize meaning-centered interventions are supported by the authors’ emphasis on “wanting to live in the moment” and rediscovering meaning via interactions with family and healthcare providers. These methods could assist medical professionals in providing holistic care that addresses relational and spiritual distress in addition to symptom management.

In conclusion, Matsumura et al. offer a crucial conceptual framework for comprehending DHD as a relational and multifaceted phenomenon. The evidence base would be further strengthened by future research that included patient views, cross-cultural comparisons, and intervention-based approaches. Research of this kind could help develop focused interventions and structured whole-person evaluation tools to help people with terminal cancer live meaningfully until death.

## References

[ref1] Borges PJ, Hernández-Marrero P and Martins Pereira S (2024) A bioethical perspective on the meanings behind a wish to hasten death: A meta-ethnographic review. *BMC Medical Ethics* 25, 23 doi:10.1017/S1478951526102028 38413954 PMC10898028

[ref2] Breitbart W, Rosenfeld B, Pessin H, et al. (2000) Depression, hopelessness, and desire for hastened death in terminally ill patients with cancer. *Jama* 284(22), 2907–2911. 11147988 10.1001/jama.284.22.2907

[ref3] Chochinov HM (2023) Intensive caring: Reminding patients they matter. *Journal of Clinical Oncology* 41(15), 2884–2887. 37075272 10.1200/JCO.23.00042 PMC10414729

[ref4] Chochinov HM, Wilson KG, Enns M, et al. (1995) Desire for death in the terminally ill. *American Journal of Psychiatry*, 152(8), 1185–1191. 7625468 10.1176/ajp.152.8.1185

[ref5] Matsumura Y, Kato H, Maetaki E, et al. (2026) Exploring the underlying structural mechanisms and whole-person perspectives on the desire for hastened death in patients with terminal cancer: A qualitative study. *Palliative and Supportive Care* 24, e100. 41943934 10.1017/S1478951526102028 PMC13166461

[ref6] Monforte-Royo C, Villavicencio-Chávez C, Tomás-Sábado J, et al. (2011) The wish to hasten death: A review of clinical studies. *Psycho-Oncology* 20(8), 795–804. 20821377 10.1002/pon.1839

[ref7] Rodríguez-Prat A, Balaguer A, Booth A, et al. (2017) Understanding patients’ experiences of the wish to hasten death: A systematic review and meta-ethnography. *BMJ Open* 7, e016659. 10.1136/bmjopen-2017-016659 PMC5640102 28965095

